# A Review on Per- and Polyfluoroalkyl Substances in Pregnant Women: Maternal Exposure, Placental Transfer, and Relevant Model Simulation

**DOI:** 10.3390/toxics11050430

**Published:** 2023-05-04

**Authors:** Yuqing Wu, Jia Bao, Yang Liu, Xin Wang, Wene Qu

**Affiliations:** School of Environmental and Chemical Engineering, Shenyang University of Technology, Shenyang 110870, China

**Keywords:** PFASs, alternative, pregnant women, placental transfer, molecular docking, machine learning

## Abstract

Per- and polyfluoroalkyl substances (PFASs) are important and ubiquitous environmental contaminants worldwide. These novel contaminants can enter human bodies via various pathways, subsequently posing risks to the ecosystem and human health. The exposure of pregnant women to PFASs might pose risks to the health of mothers and the growth and development of fetuses. However, little information is available about the placental transfer of PFASs from mothers to fetuses and the related mechanisms through model simulation. In the present study, based upon a review of previously published literature, we initially summarized the exposure pathways of PFASs in pregnant women, factors affecting the efficiency of placental transfer, and mechanisms associated with placental transfer; outlined simulation analysis approaches using molecular docking and machine learning to reveal the mechanisms of placental transfer; and finally highlighted future research emphases that need to be focused on. Consequently, it was notable that the binding of PFASs to proteins during placental transfer could be simulated by molecular docking and that the placental transfer efficiency of PFASs could also be predicted by machine learning. Therefore, future research on the maternal–fetal transfer mechanisms of PFASs with the benefit of simulation analysis approaches is warranted to provide a scientific basis for the health effects of PFASs on newborns.

## 1. Introduction

Per- and polyfluoroalkyl substances (PFASs) are a series of chemicals containing one or more perfluoroalkyl molecules (–C_n_F_2n+1_–) and have been used worldwide for the last seventy years as efficient surfactants and surface protectants [1]. It is widely known that the strong perfluoroalkyl moiety has unique characteristics, including extraordinary resistance to environmental and biological degradation, thermal and chemical stability for oxidative, photolytic, and hydrolytic reactions, and hydrophobic and oleophobic properties [2]. Due to their ubiquitous distribution globally, long-chain perfluoroalkyl carboxylic acids (PFCAs) (seven or more perfluorinated carbons) and perfluoroalkanesulfonic acids (PFSAs) (six or more perfluorinated carbons) have received widespread attention [3,4] since perfluorooctane sulfonate (PFOS) was first discovered in wildlife and even human blood 20 years ago [5,6]. Studies have revealed the potential toxicity of long-chain PFASs, leading to defects in reproduction and development, hepatotoxicity, neurotoxicity, and immunotoxicity [7,8]. PFOS, its salts, and its precursor, perfluorooctane sulfonyl fluoride (PFOSF), were restricted globally in 2009 [9]. Furthermore, other long-chain PFASs of emerging concern, perfluorooctanoic acid (PFOA) and perfluorohexane sulfonate (PFHxS), their salts, and related compounds were eliminated from production internationally in 2021 and 2022 [10,11].

Since the ban on long-chain PFAS production and usage, some replacements have been developed commercially. These alternatives have similar fluorinated chain structures, such as short-chain PFASs and polyfluorinated ethers [12,13]. Recently, these novel alternatives have been observed in human bodies, in some cases at accumulated levels, which indicates that humans have been in contact with emerging PFASs. Compared with legacy PFASs, alternatives to PFASs have higher environmental stability and mobility [14,15]. They can further migrate into the environment and widely exist and accumulate in the global environment and organisms [16,17,18,19]. The most biologically persistent PFAS is 6:2 Cl-substituted perfluoroether sulfonic acid (Cl-PFESA), which has higher hepatotoxicity and accumulation capacity than PFOS [20]. Although short-chain PFASs are generally considered easier to degrade in the environment and less toxic to humans, some studies have shown that short-chain PFASs have a similar or even greater toxicity than traditional PFASs. Notably, pregnant women exposed to PFASs can transfer these compounds from the maternal blood to the umbilical cord blood via the placenta [21,22]. Adverse risks of PFAS exposure to developing fetuses have been shown in rodent studies, possibly resulting in associations between PFAS exposure in utero and reduced birthweight [23].

The development stage of the fetus is very critical, and it is easily affected by the external environment, so we attach great importance to exposure to PFASs during fetal development. The human placenta is an important barrier to protect the fetus from the internal circulation of maternal xenobiotic compounds [24]. Epidemiological investigations showed that, among the environmental chemicals in the umbilical cord blood [25], amniotic fluid [26], and meconium, some substances can pass through the placental barrier, causing the exposure of the fetus to harmful substances in the womb. For instance, earlier studies have shown that PFASs could penetrate the placental barrier [27] and cause some adverse effects on the fetus, such as fetal growth restriction, immunosuppression, and neurotoxicity [28,29,30]. During early pregnancy, fetal organ systems are not mature, and detoxification enzymes are not fully developed [31], so fetuses are vulnerable to the impact of environmental stresses. One related mechanism may be the change in the epigenome of the fetuses, which could affect gene expression through continuous DNA methylation changes during cell division, thereby affecting the cardiac metabolic phenotype and increasing the morbidity risk [32]. In addition, another study has shown that the higher the exposure of pregnant women to PFASs during pregnancy, the higher the level of liver enzymes in children, so developmental exposure to PFASs may lead to liver damage in children [33]. Moreover, the placental transfer of PFASs might be dependent on their physical and chemical properties. A study related to PFASs in matched samples of maternal and fetal blood showed that short-chain PFASs may increase the placental transfer rate compared to long-chain congeners [34].

Chemical contaminants can cross the placental barrier by means of passive diffusion, assisted diffusion, active transport, and cytokinesis [35]. Transport proteins are important transporters of contaminants from the mother to the fetus, and the binding of certain transporters to contaminants can affect the placental transfer of contaminants [36]. In order to better explore the binding of transporters to contaminants, molecular docking techniques can be used to simulate the binding of transporters to contaminants. Molecular simulation is an effective method to explore the interaction between molecules, especially biomolecular complexes (e.g., drugs and receptors), which can obtain information on ligand and receptor binding conformations, sites, and forces [37]. Computer simulations of molecular docking techniques not only save a lot of experimental time but also provide more rapid and direct information about the biological parameters of the receptor macromolecule. A study using molecular docking to better understand the occurrence of PFASs in the human placenta and the mechanism of PFAS transfer in the human placenta revealed the binding of various types of PFASs to human serum albumin (HSA) and the affinity increasing with the length of carbon chain [38]. In addition, the binding of PFOS to the HSA was visualized using molecular docking techniques, and their binding energies and binding sites were obtained [39]. Previous research found that both linear and branched PFHxS, PFOS, and PFOA could be efficiently transported across the placenta, with the exposure levels in the order of maternal serum > cord serum > placenta [40]. In addition, another study revealed a positive association between cord PFAS levels and birth weight for male infants, as well as a positive association between branched PFOS isomers in cord blood and the gestational age of infants [41].

HSA is a globular protein that is a single-peptide chain of 585 amino acid residues. It includes 30 phenylalanine residues, 35 cysteine residues, 18 tyrosine residues, and 1 tryptophan residue. Aspartic amino acid residues exist at the N-terminal end, and leucine residues exist at the C-terminal end. A sulfhydryl group exists at position 34 of the peptide chain, and the rest are disulfide bonds, which play an important role in the maintenance of the spatial structure of HSA [42]. Alesio et al. [43] developed three models for the binding of PFASs to bovine serum albumin (BSA). All three models were able to demonstrate that PFASs can bind to the protein. Pan et al. [36] demonstrated that the binding of HSA to contaminants had an effect on placental transfer. It was shown that cord serum albumin was a positive factor in increasing the transfer efficiency, while maternal serum albumin decreased the transfer efficiency.

In recent years, some advanced tools have been used for sample classification, such as machine learning algorithms, which can be used for regression, dimension reduction, and sample classification through simple or composite models [44]. Such methods could be used to predict the physical and chemical properties of compounds [45] and have gradually been applied in a broad range of studies. Machine learning has made great progress in the past two decades. With the improvement of computer computing ability, deep learning has also made many achievements in various aspects, such as speech, natural language, and vision. The accuracy of deep learning algorithms with higher adaptability is much higher than that of classical machine learning algorithms [46]. These advantages enable artificial intelligence to play a great role in different engineering problems. The deep learning method can identify the structure and characteristics of data, such as nonlinearity and complexity, in time series prediction [47]. In previous studies, machine learning has been used to classify PFASs, which not only saves a lot of time but also makes predictions about unknown substances and helps people better understand the physical and chemical properties of these contaminants [48].

In this review, we summarized the exposure pathways of PFASs in pregnant women, the factors influencing the placental transfer efficiency, and the related mechanisms of placental transfer, based upon a review of the published literature; outlined the methods of simulation analysis for revealing the placental transfer mechanisms using molecular docking and machine learning; and finally highlighted the research emphases that need to be focused on in the future.

## 2. Methodology of Literature Sources

To obtain an overview of PFASs, the present review initially focused on PFASs in pregnant women and their placental transfer, together with a simulation analysis of placental transfer mechanisms. Reports that addressed fluorosurfactants and fluoropolymers were also included. The literature related to certain use categories was retrieved for more information on the application of PFASs.

In addition, databases and scientific studies were examined via Web of Science and PubMed. The retrieved keywords involved “emerging contaminants“, “per- and polyfluoroalkyl substances”, “PFASs”, “PFOA”, “PFOS”, “Cl–PFESA”, “short-chain”, “long-chain”, “alternative”, “substitute”, “pregnant women”, “placental transfer”, “machine learning”, “molecular docking”, “model”, and “simulation”.

The literature related to molecular docking and machine learning was summarized, compared, and analyzed. Important information about molecular docking, such as receptors, ligands, binding information, and software for molecular docking, was extracted. For the literature related to machine learning, important information such as research content, datasets, models, and validation methods was given special attention. Based upon the extraction and comparison of significant information from the literature, research areas that need to be focused on could be identified.

## 3. PFASs in Pregnant Women

### 3.1. Maternal Exposure to PFASs

The primary route of human exposure to PFASs is likely to be diet and drinking water. Previous studies have demonstrated that PFASs can enter the human body. For instance, mothers who consumed more fish could have higher concentrations of placental PFASs, since a variety of PFASs can be detected in seafood [49]. Although adult females are exposed to PFASs through indoor ambient air, house dust, and drinking water, the primary route is through the diet [50,51]. It has been suggested that the accumulation of short-chain PFASs in the human body could lead to adipogenesis, with health consequences [52]. During pregnancy, maternal PFOS are transferred through the placenta, resulting in fetal exposure to PFOS [53]. According to previous reports, branched isomers crossed the placenta more efficiently than linear isomers for both PFOS and PFOA, and the placental transfer of branched PFOS isomers increased as the branching point moved closer to the sulfonate end of the molecule [54]. The transfer efficiencies from maternal to cord sera decreased by 70% with each increasing unit of –CF_2_– chain within a PFCAs group, while those for PFOS declined by half compared to PFOA [55]. There was a significant correlation of PFAS concentrations between maternal and cord serum samples, implying the transplacental transport of PFASs. The ranking of transplacental transfer efficiency was PFOA > PFHxS > PFOS [56].

PFOS and PFOA are highly persistent in human sera, with half-lives ranging from 3.8 to 5.4 years [57], and are currently the major sources of total PFAS levels in maternal blood, umbilical cord blood, fetal blood, and even breast milk [58,59]. Postnatally, breastfed infants might be continuously exposed to PFASs through the consumption of breast milk [54]. Although the concentrations of PFASs in breast milk are one to two orders of magnitude lower than those in maternal sera [54,58], breastfeeding for 6 months significantly increased the PFAS burdens in infants. Some studies have shown that 90% of infant PFOS exposure might be attributed to breastfeeding [60]. A positive correlation between maternal PFOS concentrations and PFOS concentrations in cord blood, neonatal blood, and breast milk has been well documented in the most abundant congeners. It has been demonstrated that PFOS and PFOA concentrations in breast milk frequently exceed screening values for children’s intake of drinking water and are not limited by geographical locations. This also provides strong evidence that the main source of PFASs in infants is breast milk [61]. Previous studies have shown that one month of breastfeeding increased the concentrations of PFOS, PFOA, perfluorohexane sulfonate (PFHxS), and perfluoroheptane sulfonate (PFHpS) in infants by 3–5%, and this was independent of the prenatal PFAS concentrations. For each additional month of breastfeeding, the infant concentrations of PFOS and PFOA increased by 4% and 6%, respectively [62].

Geographic locations might also affect PFAS levels in pregnant women. Compared with cord plasma PFAS concentrations reported in other Chinese cities, the PFOS cord blood level in Beijing participants was about three-fold lower than that found in Wuhan [36,63]. Furthermore, the blood PFOA concentration in Beijing participants was eight-fold lower than that reported for Shanghai [63,64]. This was consistent with the findings of Xie et al. [65], showing lower emissions of PFOS and PFOA contaminants in Beijing compared with Wuhan and Shanghai.

**Figure 1:**
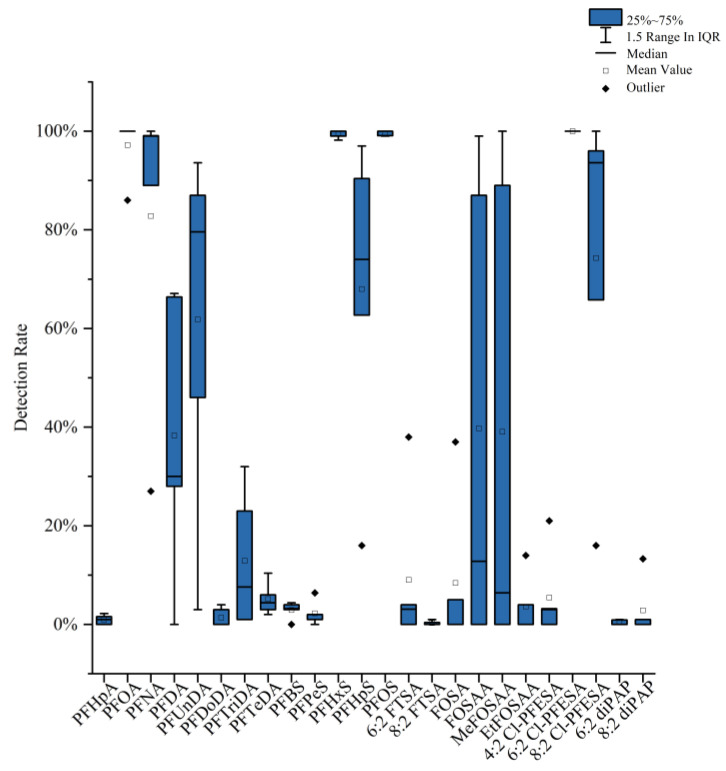
Detection rates of different PFASs in Beijing cord plasma from 1998 to 2018 (source of data: [59]).

**Figure 2:**
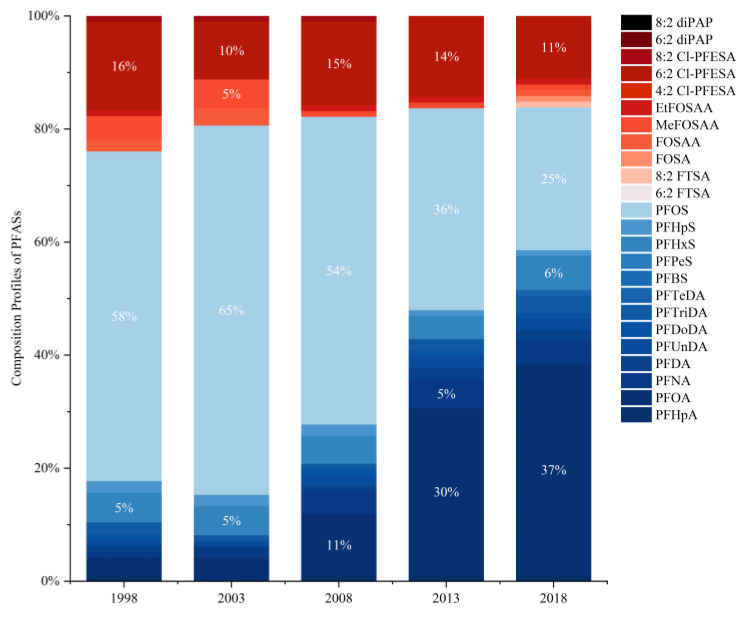
Percentage contributions of various PFASs in Beijing cord plasma from 1998 to 2018 (source of data: [59]).

Wang et al. [63] demonstrated that the levels of perfluorononanoic acid (PFNA), perfluorodecanoic acid (PFDA), perfluoroundecanoic acid (PFUnDA), PFHxS, PFHpS, and PFOS in the maternal blood were significantly and positively correlated with maternal age at delivery. Based on 650 Beijing cord plasma samples collected over a 20-year period from 1998 to 2018, another study found that the total detectable PFAS concentration in cord plasma increased from 1998 to 2003 and then decreased significantly [63]. Over time, the changing trend was more noticeable for traditional PFASs in cord plasma, such as PFOS, PFHxS, and PFOA, with PFOS showing the greatest decrease, while the trend was most remarkable for 6:2 Cl-PFESA among emerging PFASs in cord plasma. The detection rates of PFASs could give a good indication of the contaminants existing in plasma—those of PFOS, PFOA, and 6:2 Cl-PFESA were all high. It is worth mentioning that only 6:2 Cl-PFESA was detected for 100% of all the cord plasma samples (Figure 1). Moreover, the percentage contribution of 6:2 Cl-PFESA in the total concentration of detected PFASs was always second or third place compared with other PFAS congeners in cord plasma (Figure 2), which indicates that the concentration of 6:2 Cl-PFESA in human blood might no longer be negligible. Meanwhile, research has shown that the stronger binding affinity of 6:2 Cl-PFESA to HSA may contribute to its higher bioaccumulation potential than PFOS [66]. Similarly, there were comparable trends determined in maternal samples from other countries worldwide. From 1972 to 2016, the concentrations of PFHxS, PFNA, PFDA, PFUnDA, and perfluorotridecanoic acid (PFTrDA) in human milk from Stockholm increased significantly over the entire monitoring period [67]. In Australia, serum samples from subjects 0 to 60 years old and above were collected. It was found that levels of longer-chained PFDA and PFUnDA in serum samples started to decrease between 2006 and 2013, while perfluorododecanoic acid (PFDoDA) increased during the same period of time [68]. Therefore, it is important to focus on the mother-to-child transfer of emerging PFASs such as 6:2 Cl-PFESA and the potential impacts of emerging PFASs on maternal and neonatal health.

### 3.2. Placental Transfer of PFASs

PFASs are transferred from the mother to the fetus via the placenta and might be harmful to the fetus, so it is important to focus on the placental transfer of PFASs and the influencing factors. Li et al. [69] investigated the role of molecular descriptors of chemicals and placental transporters during placental transfer, and the results showed a transporter- and chemical-dependent binding affinity, indicating that molecular descriptors and placental transporters could play an important role in the placental transfer of environmental chemicals. Gao et al. [70] analyzed and calculated the placental transfer efficiencies of 21 PFASs. It was found that the placental transfer efficiency of perfluorinated alkyl carboxylic acids (PFCAs) showed a positive U-shaped trend from C4 to C13. A positive correlation between maternal body weight and PFOS transfer efficiency was also observed. Bao et al. [71] reported 20 novel PFAS congeners of four classes in human blood and placenta for the first time by analyzing maternal and umbilical cord serum and placenta samples collected from pregnant women at delivery. Furthermore, the novel PFASs were found to account for 90% of all the traditional and novel PFASs in maternal sera and even 96% of all the PFASs in placentas and umbilical cord sera. This showed that the maternal–infant transfer of emerging PFASs is also an important part that cannot be ignored. Eryasa et al. [72] observed a significant transfer of PFASs from the mother to the fetus, with transplacental transfer efficiencies ranging from a median efficiency of 36% (PFUnDA and PFDA) to 128% (branched FOSA isomer). Both functional groups and carbon chain length of different PFASs were important predictors of placental transfer and blood distribution, and transplacental transfer rates of perfluorocarboxylates and perfluorosulfonates showed a positive U-shaped relationship with carbon chain length. Liu et al. [73] investigated the isomers of PFOS, PFOA, and PFHxS in maternal and umbilical cord sera from Mianyang and Hangzhou, located in the upper and lower reaches of the Yangtze River in China, and found the isomers of PFASs in maternal and umbilical cord sera. The situation may be greatly influenced by the local production process of PFASs and the dietary habits of local residents; n–PFOS, iso–PFOS, 4m–PFOS, 1m–PFOS, n–PFOA, n–PFHxS, and br–PFHxS placental transfer efficiencies decreased significantly with increasing concentrations in maternal sera. Furthermore, Li et al. [74] analyzed novel PFASs in maternal and umbilical cord sera and found that novel PFASs accounted for a considerable proportion of total PFASs in pregnant women and could be transferred to the fetus at non-negligible concentrations. The placental transfer efficiencies of PFASs showed a positive U-shaped trend in the perfluoroalkyl carboxylic acid, perfluoroalkyl sulfonic acid, and unsaturated perfluorinated alcohol series, and those of novel PFASs were suggested to be structure-related. Overall, the carbon chain lengths, functional groups, and chemical structures of PFASs may affect the efficiencies of PFASs crossing the placental barrier [21,30].

The binding ability of PFASs to HSA might play another vital mechanistic role in the process of placental transfer. Lower placental transfer efficiencies of PFASs were associated with higher maternal HSA levels, which supports this hypothesis. It is well known that drugs and environmental contaminants penetrate the placenta mainly by means of passive diffusion. The passive diffusion of serum albumin may impede the filtration of the HSA–PFAS binding complex, and hence, only free PFASs could pass through the placenta [36].

### 3.3. Binding of PFASs to Proteins

Previous studies have shown that the carbon chain length, functional groups, and structure (linear and isomeric) of PFASs may affect the binding of HSA. However, the affinity of HSA for PFASs with different carbon chain lengths is controversial. Jones et al. [75] and Qin et al. [76] reported that the binding of bovine serum albumin (BSA) to PFASs (C4, C8, C10) increased with increasing carbon chain length. In contrast, Bischel et al. [77] found that the affinity of BSA increased from C2 to C8 and then decreased from C8 to C12, suggesting that longer carbon chains hinder binding. Gao et al. [70] analyzed the dissociation constants (K_d_) of HSA–PFAS complexes (K_d_–HP) using human sera and also found that K_d_–HP showed a positive U-shaped pattern when the carbon chain length increased. In addition, the isomeric HSA dissociation constants (K_d_) were higher and less tightly bound compared to linear PFOS and PFOA [78].

By summarizing the previous literature (Table 1), it is found that the PFASs studied were usually traditional PFASs, especially PFOS and PFOA. However, few studies have been carried out on emerging PFASs. Among the macromolecules docked with PFASs, HSA was the most studied, indicating that HSA plays an important role in placental transfer, while there are some other substances that can be molecularly docked with contaminants, such as serum albumin (SA), androgen receptor (AR), hemoglobin (Hb), human and rat liver-type fatty acid binding protein (hLFABP and rLFABP). Researchers usually use molecular docking for the study of binding energy and binding sites, and consequently, the binding of PFASs to proteins could be observed more visually, which could support the study of placental transfer mechanisms. However, the software used is relatively uniform; i.e., AutoDock is usually employed for molecular docking. In addition, placental transfer is a complex process that cannot be analyzed from a single compound, and multiple macromolecules should be studied together.

### 3.4. Simulation through Machine Learning

The placental transfer efficiencies of certain environmental chemicals have been determined in several studies by measuring concentrations in maternal and cord blood (or serum/plasma) [71]. Among various possible factors, the physicochemical properties of environmental chemicals, mainly determined by molecular descriptors, may affect their ability to diffuse across the human placental barrier or to bind to lipids, membrane transport proteins, and pharmacologically active molecules, thus affecting passive diffusion or active transport.

The physical and chemical information of chemical molecules can be described in numerical form through molecular descriptors. There are many types of molecular descriptors, such as structural indexes, topological indexes, descriptors based on two-dimensional matrices, and descriptors based on three-dimensional matrices. Molecular descriptors have now been used to predict chemical properties and material structures of chemicals [48]. Molecular descriptors are used to describe the important data on the transfer of contaminants through the placenta, and the application of molecular descriptors might not affect the prediction of placental transfer. Therefore, using molecular descriptors for model training can effectively predict the placental transfer of chemicals. Through this method, the mother-to-fetus transfer of emerging PFASs that are not fully understood might be predicted. However, it should be noted that there is no direct experiment to prove the impact of these data on placental metastasis, and using a single model to predict placental metastasis may also ignore other important factors, leading to inaccurate prediction results, which should be paid more attention in the future.

Because many PFAS components are highly resistant to degradation, PFAS contaminations could be able to persist in different environmental matrices for decades. This poses a challenge in identifying the source of detected environmental contaminations, as they may have come from decades ago or from several candidate sources at different times during previous decades. Furthermore, due to the different mobility of individual PFAS components [89], the composition of PFASs detected in the environment is similar to the original formulation released into the environment on site, but the further the detected sample is from the source, the less the observed samples often resemble the original formulation [90]. Therefore, supervised machine learning can be used to distinguish PFASs from different sources based on their composition.

The quantitative structure–activity relationship (QSAR) methodology is a useful tool to systematically analyze the information contained in the chemical structures of compounds related to existing biological data [91]. This method has been extensively applied to evaluate and predict the activity of drug molecules on therapeutic targets, as well as the toxicity risk assessment of drugs and chemicals. The QSAR model can provide information about the possible toxic effects of contaminants on the fetus. This model can be used to predict the placental metastasis of compounds. For instance, the QSAR model can be used to predict the molecular characteristics of contaminants and analyze whether contaminants can pass through the placenta. If the contaminant fails to penetrate the placenta, it might not affect the fetus [92].

Machine learning saves a lot of time in predicting unknown substances. Hyuna et al. [93] provided the first semi-supervised machine learning study for predicting structure–activity relationships for the possible bioactivities of various PFAS species. Cheng et al. [94] built machine-learning-based quantitative structure-activity relationship (QSAR) models to predict the bioactivity of PFASs. Through model prediction, the study found that most of the biologically active PFASs had perfluoroalkyl chain lengths of less than 12 and were categorized into fluorotelomer-related compounds and perfluoroalkyl acids. Lai et al. [95] used a novel machine-learning-based approach to find alternatives for the most commonly used PFAS molecules. The substitutes need to maintain their desirable chemical properties and be harmless to the organisms. By this approach, 22 promising new alternatives for PFASs were identified. Singam et al. [84] identified 29 PFASs with high potential activity against AR by screening the binding sites of PFASs to AR, and the authors concluded that these PFASs should be prioritized for biotoxicity testing. Feinstein et al. [96] used a machine learning approach to predict the acute toxicity of PFAS compounds. This approach assisted the problem of expensive in vivo experiments. The toxicity of PFASs with well-defined chemical structures was successfully predicted. Eguchi et al. [97] predicted the maternal transfer rate and molecular weight of contaminants via machine learning. Abrahamsson et al. [98] developed and tested an artificial neural network (ANN) to evaluate the extent to which small molecules, especially PFASs, could cross the placenta and partition in the cord blood. The predictions of the concentration ratio between cord and maternal blood (RCM) for PFASs suggested that 3623 compounds had a log RCM > 0, indicating preferable partitioning in cord blood.

Consequently, it is demonstrated that all of the models applied to PFASs are common in the field of machine learning, and all of them prevent overfitting by processing the dataset (Table 2). However, little information is available about machine learning studies on the placental transfer of emerging PFASs so far.

## 4. Conclusions and Future Research Emphases

In this review, we initially summarized the levels of various PFASs in pregnant women and revealed that the concentrations of PFASs in the environment in different regions were positively correlated with those of PFASs in the sera of pregnant women. Based on the levels of PFASs in cord plasma samples of pregnant women from China from 1998 to 2018, we found that the concentrations of PFASs in maternal plasma first increased and then decreased, which might be attributable to changes in international control measures for the production and usage of PFASs and related changes in the PFAS levels in the environment. Due to the updated restrictions on legacy PFASs, many alternatives of PFASs have emerged and are widely used, but the structures of some novel PFASs are not yet clear.

Secondly, we also summarized the placental transfer of PFASs and the factors influencing this process, indicating that PFASs can be transferred from the mother to the fetus via the placenta. There are many factors that affect the placental transfer of PFASs, such as carbon chain length and functional groups. Furthermore, the placental transfer efficiency of PFCAs, PFSAs, and unsaturated perfluorinated alcohols generally showed a positive U-shaped trend with increasing carbon chain length. With the production and usage of PFOS substitutes, the effects of emerging PFASs on mothers and infants would gradually become non-negligible. Previous studies have shown that emerging PFASs account for a large proportion of PFASs in mothers and infants, suggesting that maternal and fetal exposure to emerging PFASs should be focused on this in particular. However, few studies on emerging PFASs in mothers and infants are available so far.

Transporters are also a very important influencing factor, and transporters can bind to PFASs and have an effect on placental transfer. Notably, the binding of contaminants to transporters can be observed more visually using molecular docking techniques. In existing studies, PFOS is usually molecularly docked with HSA to simulate its binding mode and to obtain the binding energy and binding sites. However, limited information is available on the binding of emerging PFASs to HSA, and the proteins for docking are relatively uniform. Future studies are required to investigate the binding energy and binding sites of emerging PFASs to HSA and transporters during the placental transfer of emerging PFASs. Meanwhile, the effects of other transporters on placental transfer should also be considered.

Finally, we summarized the applications of machine learning and found that machine learning for the prediction of the physicochemical properties of compounds could be time-saving. Moreover, previous studies commonly used machine learning to predict some physical properties of PFASs and chemical properties or the structure of PFASs. Although machine learning could be employed to predict the influencing factors of maternal–fetal transfer and the efficiency of placental transfer for emerging contaminants, few studies have been implemented on PFASs so far.

As a result, future studies on the maternal–fetal transfer of emerging PFASs through the simulation analysis of molecular docking and machine learning would be warranted to reveal possible mechanisms of placental transfer in order to provide a scientific basis for the health effects of PFASs on newborns.

## Figures and Tables

**Table 1:** Previous studies on PFASs binding to different proteins.

PFASs	Target Proteins	Research Content	Software	Ref.
PFOA PFOS	HSA	Structure and energies of the binding sites	AutoDock 3.0 package	[79]
PFOS	HSA	Binding sites, binding molar ratio	–	[39]
PFBA, PFHxA, PFOA, PFDA	HSA	Binding mechanism Binding affinity	AutoDock Vina, MGLTools, Discovery Studio 3.5	[78]
PFOS, GenX	HSA	Binding sites	AutoDock 4	[80]
PFBA, PFPeA, PFHxA, PFHpA, PFOA, PFNA, PFDA, PFUdA, PFTrA, PFTeA, PFPrS, PFBS, PFPeS, PFHxS, PFHpS, PFOS, PFNS, PFDS, FOSA, N–MeFOSAA, N–EtFOSAA, 4:2 FTS, 6:2 FTS, 8:2 FTS, HFPO–DA (GenX)	HSA	Binding affinity	AutoDock Vina (v 1.1.2)	[81]
PFOA, PFOS	SA	Binding sites	AutoDock	[82]
PFOS	BSA	Binding sites	AutoDock 4.2.3	[83]
29 PFASs	AR	Binding affinity	LigPrep, Glide	[84]
PFOS	H_b_	Effects on the stability and conformation of H_b_, binding sites	Autodock 4.2.3	[85]
PFBA, PFPA, PFHxA, PFHpA, PFOA, PFNA, PFBS, PFHxS, PFOS, EEA, GenX, ADONA, 2m–PFOA, F–53, F–53B	hLFABP, rLFABP	Relative binding affinity	Autodock Vina (v1.1.2)	[86]
PFBA, PFPA, PFHxA, PFHpA, PFOA, PFNA, PFDA, PFUnA, PFDoA, PFTA, PFHxDA, PFOcDA, PFBS, PFHxS, PFOS, 6:2 FTOH, 8:2 FTOH	Liver fatty acid binding protein	K_d_ structure changes, binding strength	AutoDock 4.2	[87]
PFBA, PFHxA, PFHpA, PFOA, PFNA, PFDA, PFUnA, PFDoA, PFOcDA, PFTA, PFBS, PFHxS, PFOS, 6:2 FTOH, 8:2 FTOH, 10:2 FTOH	Thyroid hormone transport proteins	Relative potency K_d_	AutoDock 4.2	[88]

**Table 2:** Machine learning on PFASs in the literature.

Research Content	Dataset	Model	Validation	Significance	Ref.
To automatically predict the biological activity of PFASs in various human biological targets	The CF dataset, the C3F6 dataset	QSAR, unsupervised/semi-supervised machine learning models	Structural alerts were used to cross-check the validity of the predicted substructures	The first semi-supervised machine learning study of structure—activity relationships for predicting possible bioactivities in a variety of PFAS species	[93]
To predict the bioactivity of PFASs	The bioactivity information on 1012 PFASs for 26 bioassays	Logistic regression, random forests, multitask neural networks, graph convolutional models, and weave models	30% of data were used to tune hyperparameters and evaluate models	To provide valuable insight into the behavior of those chemicals and thus facilitate high-throughput screening and prioritization	[94]
To find alternatives for the most commonly used PFAS molecules	The curated EPA dataset consists of 7751 PFAS molecules	Junction tree variational autoencoder (JTVAE)	No validation set but well processed	22 promising new PFAS substitutes were identified	[95]
To classify the active and inactive PFASs for AR	The resulting dataset contained 568 active and 3934 inactive chemicals	Logistic regression, random forest, support-vector machine, k-nearest neighbors	A grid search cross-validation method was used to tune the parameters	29 PFASs had strong potential for activity against the AR	[84]
To predict acute toxicity of PFAS compounds	LDToxDB of 13,329 unique compounds of any type with oral rat LD50 measurements	RF regressor, Gaussian process (GP) regression, deep neural network, graph convolutional neural network	Five-fold cross-validation	Predicting toxicity for PFASs with a defined chemical structure	[96]
To predict the maternal–fetal transfer rates of the POPs	The Chiba University Hospital’s Delivery Unit and various other obstetric units in Japan	Principal component analysis (PCA), multiple linear regression (MLR), partial least squares regression (PLS), random forest regression (RF)	Ten-fold cross-validation	Maternal transfer rate and molecular weight, and/or lipophilicity, might be important parameters for the maternal–fetal transport of organohalogen compounds	[97]
To develop a computational approach that can be used to evaluate the extent to which small molecules can cross the placenta and partition in the cord blood	From the literature	Support-vector machine (SVM), a random forest (RF), and an artificial neural network (ANN)	Shuffle-split cross-validation with an 80/20 split	These observations have important public health implications	[98]

## Data Availability

Data availability is not applicable to this article as no new data were created in this study.
